# Mechanism of *Inonotus hispidus* in suppressing renal cell carcinoma proliferation via regulation of the PI3K/AKT/mTOR pathway

**DOI:** 10.3389/fphar.2026.1773406

**Published:** 2026-05-12

**Authors:** Yujie Wei, Hui Liu, Anxin Wang, Qi Huang, Ruyi Liu, Hao Yu, Yongxue Song, Ruining Hu, Xiuming Li

**Affiliations:** 1 Department of Urology, Affiliated Hospital of Chengde Medical University, Chengde, China; 2 Department of Prescription and TCM, School of Traditional Chinese Medicine, Chengde Medical University, Chengde, China; 3 Hebei Universities Characteristic Sericulture Application Technology Research and Development Center, Sericultural Research Institude, Chengde Medical University, Chengde, China; 4 Mulberry Silkworm Research Institute, Chengde Medical University, Chengde, China; 5 Hebei Key Laboratory of Panvascular Diseases, Affiliated Hospital of Chengde Medical University, Chengde, China

**Keywords:** *Inonotus hispidus*, molecular docking, network pharmacology, PI3K/Akt/mTOR pathway, renal cell carcinoma

## Abstract

**Ethnopharmacological Relevance:**

The medicinal relevance of *Inonotus hispidus* has been highlighted in various studies, particularly with respect to its antineoplastic, antioxidant, inflammation-modulating, and immunoregulatory capabilities. Nevertheless, its therapeutic value in renal cell carcinoma (RCC), especially the clear cell form (ccRCC), is yet to be defined.

**Aim:**

A systematic assessment of the antitumor potential and the underlying molecular mechanisms of *I*. *hispidus* against RCC by use of network pharmacology, docking simulations, and biological experiments was undertaken as the primary objective of this research.

**Materials and Methods:**

UHPLC-Q-Exactive HRMS and literature mining were used to identify bioactive constituents of *I*. *hispidus*. SwissTargetPrediction was used to predict potential molecular targets, which were then intersected with RCC-associated genes obtained in GeneCards, Therapeutic Target Database, Online Mendelian Inheritance in Man, DrugBank and PHARMGKB databases. To determine core targets, we built compound–target and protein–protein interaction (PPI) networks. GO and KEGG enrichment tools were applied for outlining the relevant pathways and biological functions to investigate the underlying biological mechanisms. Molecular docking was then implemented determine the binding potentials of active constituents with key protein targets. Antitumor activity of ethanol extract of *I. hispidus* (EEIH) was confirmed by *in vitro* experiments—CCK-8, colony formation, apoptosis, wound-healing, and Western blot—using 769-P and ACHN cell lines and *in vivo* xenograft models.

**Results:**

49 bioactive compounds and 169 overlapping targets of RCC were found. Based on target interactions, cerevisterol, withanolide, inonoterpene A and polyporusterone D were found to be major constituents. Network analysis and docking studies identified AKT1, EGFR, CTNNB1, STAT3, and BCL2 as core targets, exhibiting strong binding affinities with key compounds. The PI3K/Akt/mTOR and other cancer-related pathways were identified to be engaged in functional enrichment. EEIH treatment considerably inhibited RCC cell proliferation, colony formation, and migration, triggering apoptosis and downregulating phosphorylated Akt and mTOR. The *in vivo* antitumor efficacy of EEIH was evident in xenograft-bearing mice, where significant tumor suppression occurred in the absence of systemic toxic responses.

**Conclusion:**

The antitumor mechanism of *I. hispidus* in ccRCC involves a network of interacting molecules and pathways, particularly those regulating PI3K/Akt/mTOR signaling. The current data support further investigation into *I. hispidus* as a promising natural compound for the management of ccRCC.

## 1 Introduction

Renal cell carcinoma (RCC), accounting for the majority of kidney cancer cases, emerges due to epithelial cells lining the renal tubules. RCC accounts for over 90% of kidney cancers and is among the ten most common cancers in men (Abu-Dawas et al., 2025; Le et al., 2024). Its prevalence has been rising steadily in the last decades, especially in the case of localized-stage disease, at the rate of around 1.5% per annum. The difference in sex-based mortality is relatively small, despite the fact that RCC is more commonly diagnosed in males (Siegel et al., 2025). It is worth noting that the rate of mortality among the Native American populations is almost twice as high as the White population, which highlights the critical racial differences in healthcare outcomes (Siegel et al., 2024).

Surgical resection, radiotherapy, arterial embolization, chemotherapy, immunotherapy, and Traditional Chinese medicine (TCM) are current therapeutic strategies for RCC. Irrespective of this range of interventions, therapeutic efficacy is limited by the tumor stage, anatomical site, and patient variability, which lead to small improvements in survival. Moreover, therapeutic administration is often limited by side effects, including pyrexia, back discomfort, and gastrointestinal issues (Li et al., 2024). The current therapeutic drawbacks call for the advancement of safer and more potent treatment strategies targeting RCC.

There is growing evidence indicating that TCM has multifunctional therapeutic value in cancer management, such as alleviation of symptoms, prolongation of survival, and enhancement of quality of life. Although dysfunction of the VHL/HIF axis is a hallmark initiating event in clear cell renal cell carcinoma (ccRCC), we prioritize the PI3K/Akt/mTOR signaling pathway for several reasons: (1) this pathway serves as a critical convergence point for multiple growth factor signals, which drive tumor progression beyond initial VHL loss, thereby playing a central role in the pathogenesis of ccRCC; (2) the US Food and Drug Administration’s approval of mTOR inhibitors for the treatment of renal cell carcinoma provides strong clinical validation for the therapeutic relevance and potential for successful intervention of this pathway; (3) emerging research indicates that traditional Chinese medicine compounds exhibit preferential modulation of the PI3K/Akt/mTOR pathway, suggesting natural and potentially synergistic approaches targeting this critical signaling cascade; and (4) it is known that activation of the PI3K/Akt/mTOR pathway can lead to resistance to current standard therapies, making it a strategic and promising target for combination therapies aimed at overcoming treatment resistance and improving patient outcomes (Wang L. et al, 2022). Likewise, an aberrant stimulation of the Wnt/β-catenin pathway is also linked to enhanced tumor proliferation, stemness, and metastatic potential (Wang et al., 2018; Han et al., 2017). Some of the TCM-derived compounds have shown good anti-RCC activity by targeting these signaling axes. Gypenosides from *Gynostemma pentaphyllum* promote RCC cell apoptosis through PI3K/Akt/mTOR signaling (Liu et al., 2021), while shikonin impedes sunitinib-resistant RCC growth by activating necrosis-related proteins and downregulating Akt/mTOR (Markowitsch et al., 2022). Artesunate triggers ferroptosis and disrupts cell cycle progression in therapy-resistant RCC cells (Markowitsch et al., 2020).

*Inonotus hispidus* (Bull.: Fr.) P. Karst., also known as “Sanghuang” in traditional Chinese medicinal literature (Wu et al., 2025), is a medicinal fungus that is of importance both in its therapeutic and nutritional uses. Taxonomically, the fungus is classified under the Basidiomycota division of the Hymenochaetaceae family (Wang et al., 2023). It grows parasitically on Fraxinus mandshurica, Ulmus pumila var. mongolica, Populus, Mori Cortex, Juglans mandshurica, Malus domestica, and Sophora japonica and is widespread across several regions of China, including Northeast China, Hebei, Inner Mongolia, Beijing, Shandong, Shanxi, Ningxia, and Xinjiang (Yang S. et al, 2019). *I. hispidus* contains a wide range of pharmacologically active substances in the form of polysaccharides, sterols, phenolic acids, triterpenoids, fatty acids, amino acids, and pigments (Li and Bao, 2022a). A wide range of bioactivities, such as antitumor (Yang S. et al, 2019; Yang SD. et al, 2019; Wu et al., 2018), antioxidant (Liu et al., 2024), antibacterial (Tang et al., 2023), anti-inflammatory (Li et al., 2019), antiviral (Awadh Ali et al., 2003), neuroprotective (Wang ZX. et al, 2022) immunomodulatory (Zhang et al., 2023), and hypoglycemic are linked with these constituents. Recent research has shown its antitumor effects in several malignancies, such as breast (Zan et al., 2023; Han and Bao, 2024), cervical (Tang et al., 2023), and liver cancers (Li and Bao, 2022b). Furthermore, emerging evidence suggests that compounds derived from *I. hispidus* preferentially modulate the PI3K/Akt/mTOR signaling axis, a pathway particularly relevant to the pathogenesis and treatment resistance of clear cell renal cell carcinoma (ccRCC). There is still a significant gap in the current research on the mechanism of the impact of *I. hispidus* on renal cell carcinoma (RCC), and systematic exploration is urgently needed. The specific manifestation is that its regulatory effect on the specific immune microenvironment of ccRCC (clear cell renal cell carcinoma), such as regulatory T cells and M2 polarization of tumor associated macrophages, is not yet clear. Whether it exerts anti-tumor effects by targeting tumor stem cells or angiogenic factors (such as VEGF) remains to be verified; At the level of the PI3K/Akt/mTOR pathway, whether *I. hispidus* induces tumor cell apoptosis or enhances immune response by inhibiting PIK3CA expression, activating PTEN, or blocking mTORC1 activity, and its molecular mechanism still needs to be further elucidated; In addition, existing research lacks integrated validation of multi omics data (such as transcriptomics and proteomics) and computational biology (such as network analysis and molecular dynamics simulations), resulting in the lack of systematic experimental computational models to elucidate their multi-target regulatory effects (such as simultaneous inhibition of VEGF and PI3K) and dose-response relationships. Future research needs to focus on ccRCC specific immune regulation, molecular interventions in the PI3K/Akt/mTOR pathway, and the integration of computational and experimental techniques to reveal the multi-target action network of *I. hispidus*.

**FIGURE 1:**
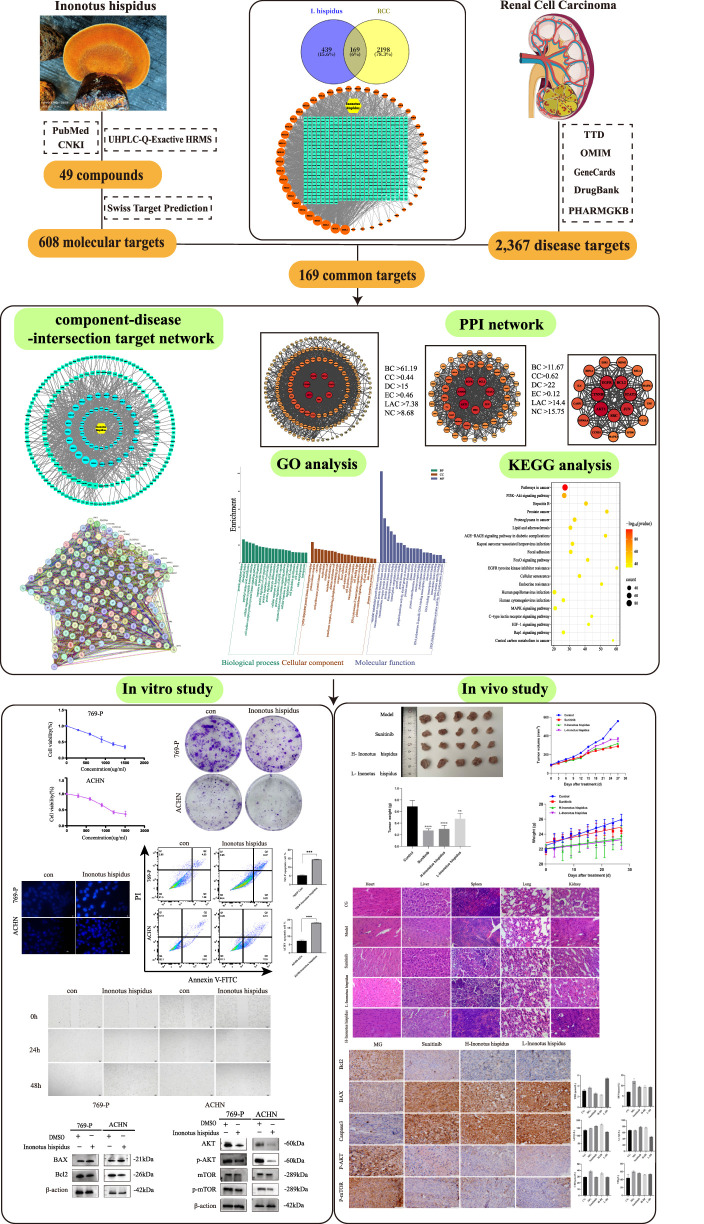
Systems-level workflow combining computational predictions and experimental analyses.

Network pharmacology, derived from systems biology, relies on computational algorithms, where the complicated drug-target-disease interactions can be explored, especially when it concerns multi-component agents, such as TCMs. It enables the detection of key targets and pathways through network construction and analysis by integrating bioinformatics tools (Hopkins, 2008). To systematically elucidate the anti-RCC potential of *I. hispidus*, this study employs an integrated multi-step approach: (1) comprehensive identification of bioactive compounds using UHPLC-HRMS-based metabolomics; (2) prediction of RCC-related therapeutic targets and signaling pathways through network pharmacology analysis; (3) validation of compound–target binding affinities via molecular docking simulations; and (4) experimental confirmation of antitumor efficacy using both *in vitro* cell models and *in vivo* xenograft systems. Network pharmacology helped identify core bioactive ingredients and anti-RCC targets associated with *I. hispidus* in this investigation. A compound–target network was built by aligning predicted targets with genes related to RCC. Protein–protein interaction (PPI) networks were built via STRING, with hub nodes determined based on topological indices like degree, closeness, betweenness centrality, eigenvector centrality, and local average connectivity (LAC). GO and KEGG enrichment methods were applied to interpret the biological roles and signaling context of the target genes. Molecular docking studies were then executed using AutoDock 4.2. The proposed biological interactions were validated experimentally *in vitro* and *in vivo*, and the methodology is diagrammed in Figure 1.

## 2 Network pharmacology and target identification

### 2.1 Identification and screening of bioactive compounds from Inonotus hispidus

To identify bioactive constituents of *I*. *hispidus*, a targeted literature search was conducted using the PubMed and CNKI databases. Compound structures were sourced from PubChem and the Traditional Chinese Medicine Systems Pharmacology Database and converted into a consistent format using ChemDraw 22.0. Pharmacokinetic properties were assessed through the SwissADME platform, with compound selection based on high gastrointestinal absorption and fulfillment of at least two of five established drug-likeness filters. Compounds were selected based on high gastrointestinal absorption (predicted by the BOILED-Egg model) and compliance with at least two of the following five drug-likeness filters: Lipinski, Ghose, Veber, Egan, and Muegge. Strict adherence to the selected filters was required; no violations were permitted. Compounds meeting these rigorous criteria were considered potentially active and were subjected to target prediction via SwissTargetPrediction. This process generated a preliminary compound–target interaction dataset for subsequent comparison with RCC-related molecular profiles.

### 2.2 Retrieval of RCC-associated targets

To assemble a comprehensive set of RCC-related targets, disease-specific gene data were retrieved from five major biomedical databases: GeneCards, Online Mendelian Inheritance in Man (OMIM), Therapeutic Target Database (TTD), DrugBank, and PharmGKB. The search strategy used a combination of terms, such as “Kidney Cancer,” “Renal cell carcinoma,” “Renal tumour,” “Renal carcinoma,” and “RCC,” with the results restricted to *Homo sapiens*. For GeneCards, only targets with a relevance score ≥37.5 were retained to prioritize high-confidence gene-disease relationships. OMIM entries were manually curated to include only those with confirmed RCC-specific phenotypes, excluding entries describing only general kidney disorders or syndromic conditions without explicit RCC association. TTD, DrugBank, and PharmGKB targets were included if they were annotated with kidney cancer or RCC-specific indications. After extraction, all targets were merged and standardized to official gene symbols using the UniProt database. A deduplication process was performed to remove redundancies, and targets appearing in ≥2 databases were prioritized as high-confidence RCC-associated genes for subsequent analysis. This multi-database integration strategy yielded 2,367 unique RCC-related targets for network construction.

### 2.3 Compound–target interaction network construction

Evenn (Yang et al., 2024) was utilized to detect the intersection between RCC-related genes and predicted targets of compounds from *I. hispidus*, with the aim of uncovering possible mechanisms of action. The overlapping targets were visualized through a Venn diagram to identify similar molecular components. Using Cytoscape 3.9.1, the shared targets and their respective compounds were assembled into a network diagram illustrating compound–target interactions.

### 2.4 PPI network construction

To examine inter-target functional connectivity, the overlapping genes were analyzed through the “Multiple Proteins” feature in STRING (v12.0), resulting in the generation of a PPI network. Protein interactions specific to *H. sapiens* were selected, with a confidence score threshold of at least 0.7, and isolated nodes were not included. The data regarding the interactions were loaded into Cytoscape 3.9.1, where the interactions were visualized and topological metrics were analyzed by use of the CytoNAC plugin. Node centrality within the network was quantified using several topological parameters, including degree, betweenness, closeness, eigenvector metrics, LAC, and overall centrality. Core targets that might be engaged in the therapeutic activity of *I. hispidus* were selected based on a two-tier screening process, in which only nodes with a value greater than the median of all centrality metrics were retained.

### 2.5 GO and KEGG analyses

Metascape-based enrichment analysis was used to characterize biological functions and pathways of the shared targets, with *H. sapiens* being a reference organism, in order to guide enrichment calculations. Through GO enrichment, targets were grouped into biological processes (BPs), cellular components (CCs), and molecular functions (MFs), KEGG enrichment provided insights into signaling pathways that might be implicated in the therapeutic effect of *I. hispidus* on RCC. A bar chart was generated to illustrate the top 20 GO and KEGG terms with the greatest enrichment significance by use of Bioinformatics (https://www.bioinformatics.com.cn), which helps understand the molecular processes behind the RCC modulation.

### 2.6 Molecular docking assessment

Key compound–target interactions were validated by molecular docking simulations, in order to determine the binding affinity and spatial compatibility. In accordance with validated molecular docking practices, interactions yielding binding energies at or below −5.0 kcal/mol were deemed energetically favorable. A network-based selection identified five hub compounds with the greatest number of target interactions, which were then considered as candidate ligands.

Proteins used in docking were acquired from the Protein Data Bank (PDB) repository. The preparation procedure involved removing water molecules and non-protein molecules, followed by the minimization of energy by ChemDraw 3D 23.1.1. The optimized structures were stored in PDBQT format. Ligand structures were acquired from PubChem and processed in ChemDraw before being prepared using AutoDock Tools 4.2.6, which included hydrogenation, charge assignment, and torsion parameter specification. Docking simulations were executed in AutoDock 4.2.6, with grid boxes defined to encompass the active binding regions of each target. The Lamarckian genetic algorithm was employed with 50 independent runs to explore binding conformations and identify optimal interaction poses. For structural interpretation, PyMOL and Discovery Studio 2019 were utilized to visualize docking conformations.

### 2.7 Extraction and UHPLC-Q-exactive HRMS analysis of *Inonotus hispidus*

To profile the chemical constituents of *I*. *hispidus*, fruiting bodies were provided by the Sericulture Institute of Chengde Medical College (Hebei Province, China) and stored under controlled laboratory conditions. The samples were oven-dried and pulverized into fine powder prior to extraction. The powder was first subjected to aqueous extraction, performed in three sequential cycles at 80 °C for 2 h each. The extraction solvent-to-powder ratios were 1:6 (w/v) for the first round using 3000 mL of water, and 1:4 (w/v) for the subsequent two rounds, each with 2000 mL of water. The remaining residue was then extracted using 75% ethanol under reflux conditions, also in three cycles of 2 h each at 60 °C, employing the same solvent ratios as the aqueous extraction. To prepare for chemical analysis, the ethanol and aqueous extracts were blended, concentrated under vacuum conditions, and subjected to freeze-drying.

Reconstitution of the dry extract in a compatible solvent was followed by analysis via ultra-high-performance liquid chromatography (UHPLC) interfaced with a Q-Exactive Orbitrap mass spectrometer. Separation was conducted using a BEH C18 column (ACQUITY UPLC, 2.1 mm × 100 mm, 1.7 μm), thermostatted at 40 °C, on a Vanquish UHPLC platform. Mobile phase solvents—0.01% aqueous formic acid (A) and acetonitrile (B)—were pumped at 0.3 mL/min. The LC gradient was initiated with 5% B, ramped up to 95% B within 2 min, gradually decreased to 10% B over the next 48 min, held constant at 10% for 5 min, then increased back to 95% B over the final 5 min. MS data were acquired on a Q-Exactive Orbitrap instrument using electrospray ionization (ESI) in dual polarity modes to maximize compound coverage. High-resolution full-scan MS data were acquired for subsequent compound identification and structural elucidation. Compounds were identified by matching accurate mass measurements (mass error ≤5 ppm) and MS/MS fragmentation patterns against public spectral databases (GNPS, MassBank, METLIN, NIST).

## 3 Experimental validation

### 3.1 Materials and reagents

The RCC cell lines 769-P and ACHN, sourced from Wuhan Pricella Biotechnology Co., Ltd., were used in this study. For protein expression analysis, the following primary antibodies were utilized: Bcl-2 (124) (15071S; Cell Signaling Technology, CST), Bax (2772S; CST), and Caspase-3 polyclonal antibody (PA5-77887; Thermo Fisher). Additional antibodies included Akt (ST05-09), phosphorylated Akt (p-Akt; SD08-12), mTOR (SU30-00), phosphorylated mTOR (p-mTOR; A5D5), and GAPDH (clone 14C10; CST).

### 3.2 Cell culture

Both 769-P and ACHN RCC lines were cultured in media (RPMI-1640 for 769-P, MEM for ACHN; Gibco, China) containing 10% fetal bovine serum (BioInd) and 1% penicillin–streptomycin (BasalMedia). Incubation conditions included 5% carbon dioxide, 37 °C temperature, and sustained humidity for proper cell culture maintenance.

### 3.3 CCK-8 assay

Cell viability in response to EEIH treatment was quantified using the CCK-8 assay. Cells (769-P and ACHN) were cultured in 96-well plates (8 × 10^3^ cells/well in 100 μL medium) and incubated for 24 h to permit adhesion. EEIH was then added at final concentrations of 0, 300, 600, 900, 1200, and 1500 μg/mL (10 μL per well). Each well was exposed to 100 mL of CCK-8 reagent (Dojindo Laboratories) after 48 h of treatment and incubated over 2 h under standard conditions (37 °C, 5% CO_2_). The absorbance readings at 450 nm were obtained using a FLUOstar Omega instrument (BMG Labtech). Viability percentages were derived from the equation: [(A_extracts_ - A_blank_)/(A_DMSO_ - A_blank_)] × 100%.

### 3.4 Colony formation assay

RCC cells underwent a colony formation assay after the exposure to EEIH to quantify their ability to form colonies over time. The assay commenced with the seeding of ACHN and 769-P cells (5 × 10^3^ cells/well) in 12-well plates, which then underwent 24-h incubation at 37 °C in 5% CO_2_ to enable attachment. Afterward, EEIH was administered at specific concentrations and incubated for 14 days to enable the development of colonies. For visualization of formed colonies, a fixation step using 4% paraformaldehyde for 15 min was followed by staining with 0.1% crystal violet. Colonies were counted by use of ImageJ software based on digital images.

### 3.5 Hoechst staining assay

A 24-h exposure to 1000 μg/mL EEIH was administered to 769-P and ACHN cells. Post-treatment, cells were rinsed with Buffer A and fixed for 10 min in a 4% formaldehyde solution. A 10-min staining step at room temperature (RT) was performed using Hoechst 33258 (diluted 1:10; Jiangsu KeyGEN BioTECH Corp., Ltd.). Fluorescent nuclei were visualized at 340 nm excitation by use of a fluorescence microscope.

### 3.6 Flow cytometry assays

769-P and ACHN cells underwent treatment with Fas ligand (FasL)-neutralizing antibody (10 μg/mL) at 37 °C as a pretreatment step, after which EEIH (20 μg/mL) was added for 24-h treatment. Afterward, phosphate-buffered saline (PBS) wash was performed before cell detachment and centrifugation (300 × g for 5 min). Pellets of cells (1 × 10^5^ cells) were carefully in 1× Binding Buffer at 100 μL volume. Apoptotic status was evaluated using Annexin V-FITC and propidium iodide (PI). Staining involved 15 min with Annexin V-FITC, followed by 5 min with PI, both protected from light. Flow cytometric analysis was implemented immediately, and apoptotic populations were counted by use of standard gating strategies.

### 3.7 Wound-healing assay

Six-well plates were seeded with cells and left to grow until reaching full confluency. Cell monolayers were wounded by drawing a straight line through the culture surface by use of a sterile 200 μL pipette tip. The cultures were then incubated in EEIH-containing, serum-deprived medium to assess cell migration. Microscopic images of the scratched region were obtained at 0, 24, and 48 h following treatment using an inverted microscope. These were analyzed in ImageJ to determine the area of wound closure, calculated as: [(initial wound area − remaining wound area)/initial area] × 100%.

### 3.8 Western blot analysis

A 24-h treatment with EEIH or DMSO was applied to 769-P and ACHN cells cultured in 12-well plates. Cell lysis was then carried out using RIPA lysis buffer (Beyotime Biotechnology, Jiangsu, China). Total protein concentrations were quantified by use of a BCA assay kit (Beyotime Biotechnology). Protein extracts (20 μg/lane) were resolved via 10% sodium dodecyl sulfate-polyacrylamide gel electrophoresis (SDS-PAGE) and transferred to polyvinylidene difluoride (PVDF) membranes at 250 mA for 2 h. Blocking in 5% TBST-dissolved skim milk was conducted for 60 min (RT), followed by an overnight primary antibody incubation at 4 °C. Three TBST washes preceded a 1-h incubation with HRP-conjugated secondary antibodies at RT. Enhanced chemiluminescence was used for signal development, and resulting protein bands visualized on the ChemiDoc MP (Bio-Rad).

### 3.9 Animal modeling, grouping, and intervention

An RCC xenograft model was constructed in SPF-grade female BALB/c nude mice (6–8 weeks old, 22 ± 2 g) to test the antitumor function of *I. hispidus in vivo*. After being housed for a one-week acclimation period under specific pathogen-free conditions, mice purchased from Vital River Laboratory Animal Technology Co., Ltd. (Beijing, China) were randomly grouped into five sets of five individuals. A single-cell suspension of ACHN renal carcinoma cells (5.0 × 10^6^ cells/mL in saline) was subcutaneously injected into the right axillary region at a volume of 0.15 mL per mouse. When tumor volumes averaged near 100 mm^3^, treatments were initiated. The groups were as follows: tumor-free control (no tumor implantation), Model group (tumor-bearing, vehicle-treated), Positive control group (sunitinib, 40 mg/kg/day), EEIH low-dose group (200 mg/kg/day), and EEIH high-dose group (400 mg/kg/day). All treatments were administered once daily by oral gavage (0.015 mL/g body weight). At intervals of 3 days, tumor dimensions and body weights were monitored. Volumes were computed using the formula: V = 0.5 × length × width^2^. After completing 27 days of treatment, mice were euthanized using cervical dislocation. Tumors were excised and weighed, and heart, liver, spleen, lungs, and kidneys were sampled for hematoxylin and eosin (H&E) staining.

### 3.10 H&E analysis and immunohistochemistry

After fixation in 10% neutral-buffered formalin, harvested organs were processed for paraffin embedding and sectioned at 5 mm in order to evaluate organ toxicity and tissue-level effects of EEIH treatment. To examine tissue histopathology, sections were stained with hematoxylin and eosin, with hematoxylin staining nuclei and eosin counterstaining cytoplasmic components. As part of the immunohistochemical workflow, paraffin-embedded tumor sections underwent deparaffinization, rehydration, and antigen retrieval treatment. Hydrogen peroxide was applied to block native peroxidase activity prior to incubation with primary antibodies against apoptosis- and signal transduction-related targets. After application of HRP-conjugated secondary antibodies, 3,3′-diaminobenzidine (DAB) was used for chromogenic detection. Slides were counterstained with hematoxylin, dehydrated, mounted, and examined under a light microscope.

### 3.11 Serum biochemical analysis of liver and kidney function

At the end of the treatment period, whole blood was collected from mice prior to tissue harvest. Blood samples were allowed to clot at room temperature and then centrifuged to obtain serum. Serum biochemical parameters, including aspartate aminotransferase (AST), alanine aminotransferase (ALT), blood urea nitrogen (BUN), creatinine (CRE), total protein (TP), and total bilirubin (TBIL), were measured using commercial assay kits following the manufacturers’ instructions (or an automated biochemical analyzer, if applicable). These indices were used to evaluate potential hepatotoxicity and nephrotoxicity associated with EEIH administration.

### 3.12 Statistical analysis

All statistical analyses were performed using GraphPad Prism 9.0. Data are presented as mean ± SD. Normality and variance homogeneity were assessed using Shapiro-Wilk and Levene’s tests, respectively, with non-parametric tests applied when assumptions were violated. *In vitro* data were analyzed by one-way ANOVA with Tukey’s post-hoc test or unpaired two-tailed Student’s t-test. *In vivo* tumor volume and body weight were analyzed by two-way repeated measures ANOVA with Bonferroni correction; final tumor weight was analyzed by one-way ANOVA with Tukey’s correction. The sample size (n = 5 mice per group) was determined based on pilot study effect sizes, power analysis (>80% power at α = 0.05) and 3R ethical principles. Statistical significance was defined as p < 0.05 (two-tailed), with exact p-values reported.

## 4 Results

### 4.1 Active compound-targets network analysis

**FIGURE 2:**
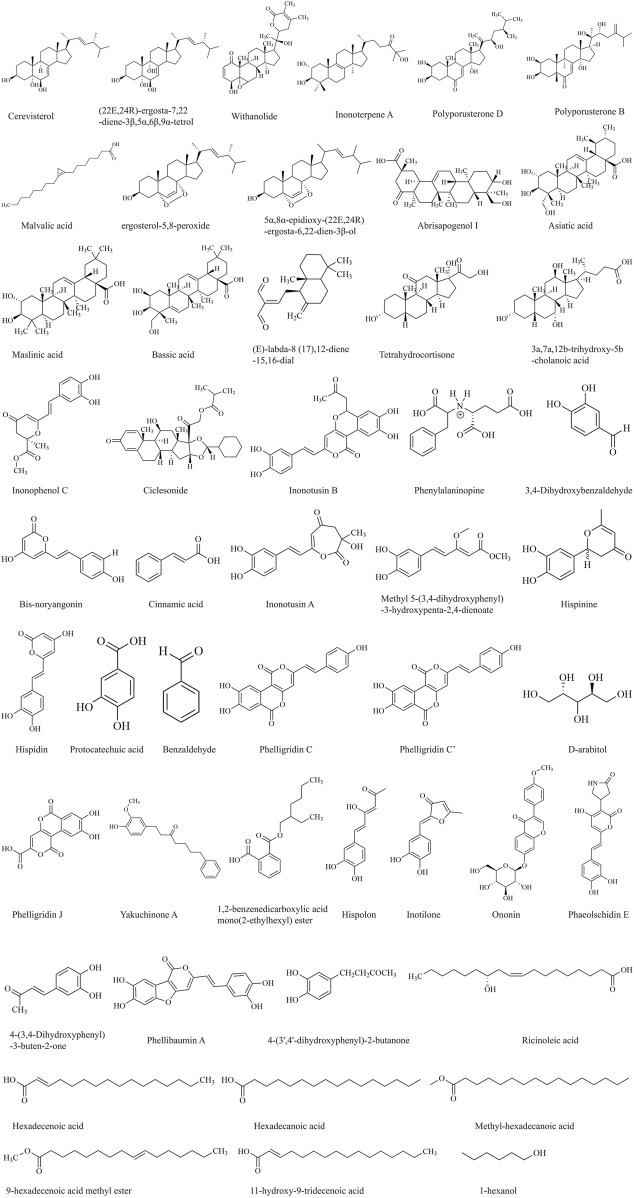
Molecular formulas of 49 *Inonotus hispidus*.

To identify potential therapeutic constituents of *I. hispidus* against RCC, 49 bioactive compounds were selected based on high gastrointestinal absorption and compliance with at least two established drug-likeness filters. These compounds were retrieved through comprehensive literature mining from the PubMed and CNKI databases, and their molecular formulas were drawn using ChemDraw (Figure 2). Target prediction using SwissTargetPrediction yielded 608 associated protein targets. Compound–target relationships were graphically represented with the aid of Cytoscape 3.9.1 for network visualization (Figure 4B), comprising 658 nodes (1 drug node, 49 compound nodes, and 608 target nodes) and 2,463 edges. Orange circular nodes represent bioactive compounds, with node size corresponding to degree centrality, while green square nodes denote predicted molecular targets. The highest degree values were obtained in several compounds, including cerevisterol, (22E,24R)-ergosta-7,22-diene-3β,5α,6β,9α-tetrol, withanolide, Inonoterpene A, and polyporusterone D. Table 1 shows detailed compound data.

**TABLE 1:** Active ingredients of 49 *Inonotus hispidus*.

MOL ID	Compound	Average shortest path length	Betweenness centrality	Closeness centrality	Degree	References
MOL1	cerevisterol	2.595129376	0.076196405	0.385337243	109	Wang et al. (2022b)
MOL2	(22E,24R)-ergosta-7,22-diene-3β,5α,6β,9α-tetrol	2.598173516	0.087483702	0.384885764	108	Wang et al. (2022b)
MOL3	withanolide	2.601217656	0.092902934	0.384435342	107	Li and Bao (2022a)
MOL4	Inonoterpene A	2.604261796	0.04795027	0.383985973	106	Wang et al. (2022b)
MOL5	Polyporusterone D	2.607305936	0.068780971	0.383537653	105	Zan et al. (2023)
MOL6	Polyporusterone B	2.610350076	0.077894162	0.383090379	104	Zhijun and Haiying (2022)
MOL7	yakuchinone A	2.613394216	0.127866504	0.382644147	103	Han and Bao (2024)
MOL8	1,2-benzenedicarboxylic acid mono (2-ethylhexyl) ester	2.616438356	0.124577165	0.382198953	102	Wang et al. (2022b)
MOL9	malvalic acid	2.616438356	0.048061086	0.382198953	102	Han and Bao (2024)
MOL10	ricinoleic acid	2.616438356	0.044292473	0.382198953	102	Han and Bao (2024)
MOL11	ergosterol-5,8-peroxide	2.628614916	0.064080037	0.380428489	98	Wang et al. (2022b)
MOL12	11-hydroxy-9-tridecenoic acid	2.628614916	0.060111554	0.380428489	98	Han and Bao (2024)
MOL13	5α,8α-epidioxy-(22E,24R)-ergosta-6,22-dien-3β-ol	2.643835616	0.059180085	0.378238342	93	Zhijun and Haiying (2022)
MOL14	Abrisapogenol I	2.671232877	0.02023839	0.374358974	84	Huo et al. (2022)
MOL15	hexadecanoic acid	2.674277017	0.073920432	0.37393284	83	Zan et al. (2023), Zhijun and Haiying (2022)
MOL16	Maslinic acid	2.695585997	0.020248586	0.370976849	76	Huo et al. (2022)
MOL17	Asiatic acid	2.713850837	0.015973196	0.36848009	70	Huo et al. (2022)
MOL18	Bassic acid	2.719939117	0.017543087	0.367655288	68	Li and Bao (2022a)
MOL19	9-hexadecenoic acid methyl ester	2.722983257	0.025637785	0.367244271	67	Zhijun and Haiying (2022)
MOL20	tetrahydrocortisone	2.741248097	0.039336431	0.364797335	61	Li and Bao (2022a)
MOL21	inonophenol C	2.750380518	0.059996849	0.363586054	58	Wang et al. (2022b)
MOL22	Hispolon	2.771689498	0.034947073	0.360790774	51	Zhijun and Haiying (2022)
MOL23	4-(3′,4′-dihydroxyphenyl)-2-butanone	2.771689498	0.043511895	0.360790774	51	Wang et al. (2022b)
MOL24	4-(3,4-Dihydroxyphenyl)-3-buten-2-one	2.792998478	0.030797079	0.358038147	44	Zhijun and Haiying (2022)
MOL25	(E)-labda-8 (17),12-diene-15,16-dial	2.802130898	0.033982077	0.356871266	41	Han and Bao (2024)
MOL26	Methyl-hexadecanoic acid	2.811263318	0.016813379	0.355711965	38	Zan et al. (2023)
MOL27	ciclesonide	2.823439878	0.020150711	0.354177898	34	Li and Bao (2022a)
MOL28	3a,7a,12b-trihydroxy-5b-cholanoic acid	2.829528158	0.004075749	0.353415815	32	Li and Bao (2022c)
MOL29	cinnamic acid	2.832572298	0.016523035	0.353036002	31	Wang et al. (2022b)
MOL30	protocatechuic acid	2.844748858	0.018555005	0.35152488	27	Shu-Dong et al. (2019)
MOL31	Inotilone	2.847792998	0.022108209	0.351149118	26	Zan et al. (2023)
MOL32	ononin	2.847792998	0.024659903	0.351149118	26	Han and Bao (2024)
MOL33	Inonotusin B	2.850837139	0.007145771	0.350774159	25	Zhijun and Haiying (2022)
MOL34	phenylalaninopine	2.866057839	0.028148656	0.348911312	20	Wang et al. (2022b)
MOL35	3,4-Dihydroxybenzaldehyde	2.872146119	0.006809176	0.348171701	18	Zhijun and Haiying (2022)
MOL36	1-hexanol	2.875190259	0.005580209	0.34780307	17	Zhijun and Haiying (2022)
MOL37	methyl 5-(3,4-dihydroxyphenyl)-3-hydroxypenta-2,4-dienoate	2.878234399	0.005988319	0.347435219	16	Wang et al. (2022b)
MOL38	Hexadecenoic acid	2.887366819	0.001070746	0.346336321	13	Zan et al. (2023)
MOL39	Inonotusin A	2.899543379	0.008687014	0.34488189	9	Zhijun and Haiying (2022)
MOL40	hispinine	2.902587519	8.58E-04	0.344520189	8	Wang et al. (2022b)
MOL41	Benzaldehyde	2.911719939	0.001204594	0.343439624	5	Zhijun and Haiying (2022)
MOL42	Phaeolschidin E	2.911719939	0.00624098	0.343439624	5	Zan et al. (2023)
MOL43	Bis-noryangonin	2.914764079	0.003216263	0.34308094	4	Zhijun and Haiying (2022)
MOL44	Hispidin	2.914764079	0.001636819	0.34308094	4	Zhijun and Haiying (2022)
MOL45	Phellibaumin A	2.917808219	0.003061029	0.342723005	3	Zan et al. (2023)
MOL46	Phelligridin C	2.917808219	4.18E-04	0.342723005	3	Zan et al. (2023), Zhijun and Haiying (2022)
MOL47	Phelligridin C′	2.917808219	4.18E-04	0.342723005	3	Zhijun and Haiying (2022)
MOL48	D-arabitol	2.917808219	0.003694769	0.342723005	3	Wang et al. (2022b)
MOL49	Phelligridin J	2.920852359	1.79E-04	0.342365816	2	Zan et al. (2023), Zan et al. (2023)

**FIGURE 3:**
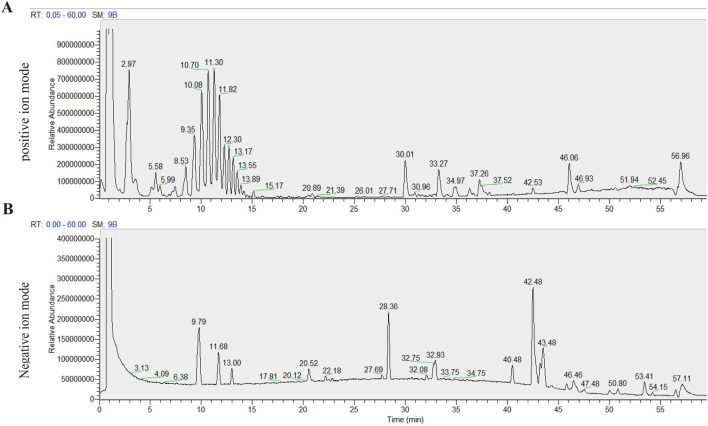
Total ion chromatograms of *Inonotus hispidus* in electrospray ionization positive **(A)** and negative **(B)** ion modes acquired by UHPLC-Q-Exactive Orbitrap high-resolution mass spectrometry.

In order to experimentally validate the predicted compounds, the UHPLC-Q-Exactive HRMS analysis of EEIH was implemented. Total ion chromatograms (TICs) obtained under positive and negative ion modes showed different chromatographical peaks representing fatty acids, terpenoids, and polyphenolic compounds. It took a 60-min running time to complete the analysis (Figure 3), and mass spectrometry data of the identified constituents are listed in Table 2.

**TABLE 2:** Mass spectrometry data table of constituents in *Inonotus hispidus* based on UHPLC - MS analysis.

tR (min)	Experimental mass (m/z)	Molecular formula	Fragmentation (m/z)			Compound
9.79	177.05571	C_10_H_10_O_3_	121.0491	135.044	149.06	Osmundacetone
11.68	217.05063	C_12_H_10_O_4_	128.0341	173.0924	181.071	Inotilone
13.00	489.08271	C_26_H_18_O_10_	191.0345	245.0452	269.0462	3,14′-bihispidinyl
17.81	379.04594	C_20_H_12_O_8_	217.1191	245.113	159.1301	Phelligridin D
20.52	543.12967	C_30_H_24_O_10_	238.0821	287.5542	296.1048	Phaeolschidin A ion
20.52	363.05102	C_20_H_12_O_7_	112.9844	135.044	155.1068	Phaeolschidin C
22.18	503.09837	C_27_H_20_O_10_	213.0549	245.0449	257.0458	SCHEMBL8676491
28.36	559.16097	C_31_H_28_O_10_	245.0454	269.1191	313.1084	Phaeolschidin B
32.08	485.32724	C_30_H_46_O_5_	290.2484	318.2792	362.269	Abrisapogenol I
32.08	485.32724	C_30_H_46_O_5_	325.2532	359.6371	431.3057	Bassic acid
32.75	363.21769	C_21_H_32_O_5_	217.0134	241.1269	269.0096	Tetrahydrocortisone
32.93	295.22786	C_18_H_32_O_3_	237.186	267.9725	277.2177	Hydroxy-octadecadienoic acid
33.75	297.24350	C_18_H_34_O_3_	115.0025	133.013	181.071	Ricinoleic acid
34.75	293.21221	C_18_H_30_O_3_	249.2224	275.2033	293.2128	Hydroxy-octadecatrienoic acid
42.48	253.21730	C_16_H_30_O_2_	209.1544	219.5377	253.1205	Hexadecenoic acid
43.48	279.23295	C_18_H_32_O_2_	225.9843	244.0614	262.073	Hexadecanoic acid
46.46	281.24860	C_18_H_34_O_2_	243.7909	257.8292	281.2488	Octadecenoic acid
47.48	281.24860	C_18_H_34_O_2_	129.091	139.3854	183.1386	Oleic acid
50.80	283.26425	C_18_H_36_O_2_	219.8441	239.1652	265.1448	Octadecanoic acid
53.41	355.10345	C_16_H_20_O_9_	112.9843	171.0054	241.1206	Gentiopicroside
53.41	391.30063	C_28_H_40_O	170.1544	229.1555	271.1667	(22E,24x)-Ergosta-4,6,8,22-tetraen-3-one
54.15	391.30063	C_28_H_40_O	229.1555	271.1667	301.1781	Ergosta-4,6,8 (14),22-tetraen-3-one

**FIGURE 4:**
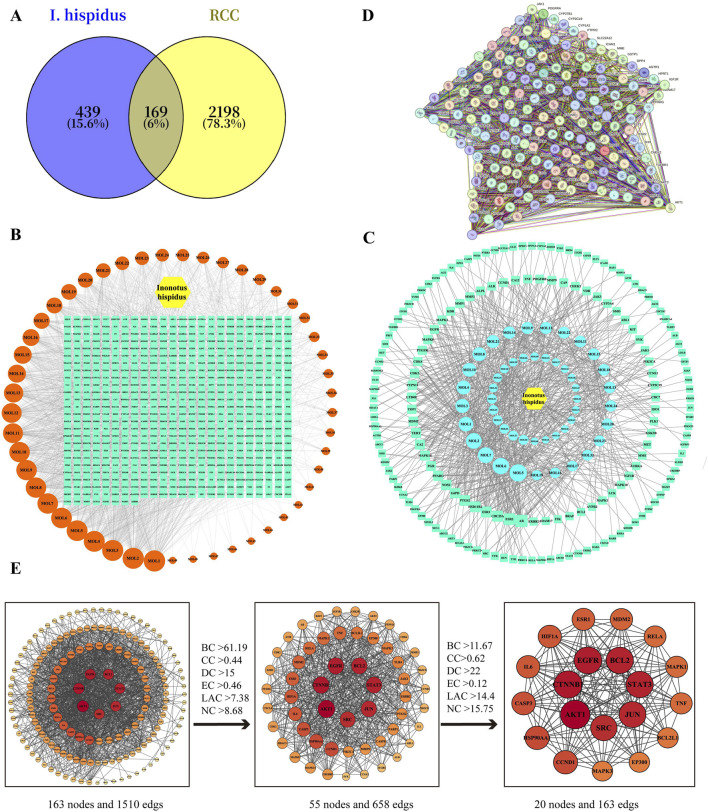
Analysis of drugs-intersection targets. **(A)** Venn diagram (The intersection targets of *Inonotus hispidus* and RCC). **(B)** Drug-component target network diagram. (The yellow hexagon represents Inonotus hispidus, the orange circle represents a component, and the green square represents a target). **(C)** Drug component-disease-intersection target network diagram. (The blue circle represents the pharmaceutical component, and the green diamond represents the intersection target.). **(D)** PPI network from STRING database of *Inonotus hispidus* and RCC intersection targets (Nodes represent proteins, edge represents protein-protein association). **(E)** Construct a PPI network diagram via Cytoscape and screen out core targets.

Simultaneously, five disease-related databases, namely, GeneCards, TTD, OMIM, DrugBank, and PharmGKB were searched, yielding 2,367 RCC-associated targets. These were compared with the 608 predicted targets of *I. hispidus* after integration and deduplication. Venn diagram analysis revealed 169 overlapping genes (Figure 4A), representing potential molecular intersections between the compound set and RCC. A refined compound–target–disease interaction network was then constructed (Figure 4C), comprising 220 nodes—1 drug node, 1 disease node, 49 compound nodes, and 169 shared target nodes—linked by 762 edges.

### 4.2 PPI network construction and core target screening

To identify key regulatory proteins potentially mediating the anti-RCC effects of *I*. *hispidus*, the 169 overlapping targets were input into the STRING database to generate a PPI network (Figure 4D). After filtering out six nodes lacking interaction data, the final network included 163 proteins connected by 1,510 edges, representing high-confidence functional associations (Figure 4E). Topological analysis was conducted using the CytoNCA plugin within Cytoscape 3.9.1. Six centrality metrics were calculated—degree, betweenness, closeness, eigenvector centrality, LAC, and overall network centrality to assess the structural importance of each node within the network. Nodes with values greater than the median in ≥4 of 6 metrics were retained as core targets. The median-based threshold was selected in this study, which is well established to be able to retains sufficient nodes for meaningful biological interpretation while filtering out peripheral nodes with minimal network influence. This analytical process yielded 20 core targets, which are detailed in Table 3.

**TABLE 3:** 20 core targets screened by PPI network.

Name	Betweenness	Closeness	Degree	Eigenvector	LAC	Network
AKT1	168.8825	0.870968	46	0.223546	21.17391	40.87665
CTNNB1	118.2432	0.830769	43	0.213073	20.55814	36.75668
EGFR	133.3308	0.818182	42	0.204148	19.52381	34.72053
STAT3	99.91885	0.80597	41	0.208191	21.02439	35.15915
BCL2	96.75563	0.80597	41	0.20859	20.87805	34.66569
JUN	77.59067	0.794118	40	0.208145	21.5	34.4336
SRC	98.04609	0.782609	39	0.193111	19.48718	32.47326
HSP90AA1	60.02993	0.72973	34	0.173001	17.41176	24.77406
CCND1	51.61868	0.72973	34	0.178902	18.70588	26.02022
CASP3	58.59114	0.72	33	0.170139	17.93939	25.37292
IL6	35.19866	0.710526	32	0.173898	19.6875	26.00045
HIF1A	35.23531	0.710526	32	0.176766	19.625	25.2236
ESR1	38.6781	0.701299	31	0.16801	18.06452	23.01101
MDM2	40.86702	0.692308	30	0.154333	17.13333	23.24628
MAPK1	33.86474	0.683544	29	0.15633	16.41379	20.07854
TNF	38.39288	0.683544	29	0.154042	17.72414	22.65364
RELA	33.68727	0.683544	29	0.157574	16.96552	20.78225
BCL2L1	30.46888	0.666667	27	0.145048	15.55556	18.72334
EP300	23.59964	0.658537	26	0.139088	16.30769	19.84211
MAPK3	18.32301	0.658537	26	0.148542	16.76923	18.78989

### 4.3 GO and KEGG pathway enrichment analysis

To investigate the functional roles of the 20 core targets identified in the PPI network, GO and KEGG enrichment analyses were performed using Metascape.

**FIGURE 5:**
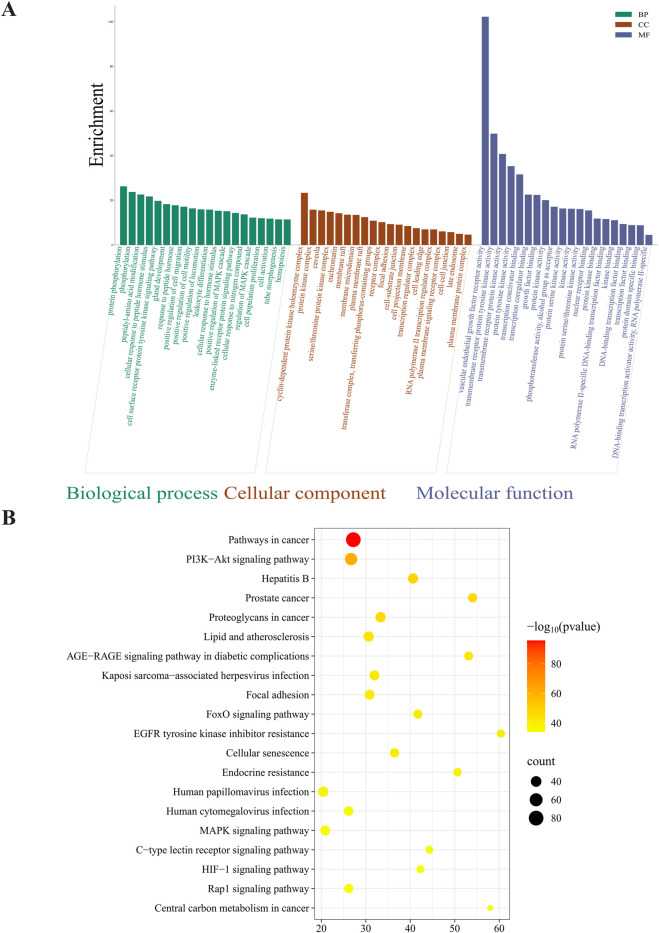
**(A)** GO analysis of drug-disease intersection targets (biological processes (green), cellular components (orange), and molecular functions (purple). **(B)** KEGG analysis of intersection targets.

GO analysis yielded 2,371 enriched terms, subdivided into 2,064 BP, 111 CC, and 196 MF terms. The top 20 most statistically significant terms from each category were selected and visualized as functional micro-networks (Figure 5A). BP terms were enriched for pathways involving phosphorylation of proteins, modulation of transduction signals, and cellular reactions to mitogenic signals. CC categories included cytoplasmic regions, the plasma membrane surface, and membrane-bound protein assemblies. Molecular functions included ATP binding, protein kinase activity, and transmembrane receptor activity. The most prominent among these were t Among the pathways identified, PI3K-Akt signaling, EGFR tyrosine kinase inhibitor resistance, and proteoglycan-mediated oncogenic mechanisms were the most biologically relevant, alongside general cancer-associated pathways (Figure 5B; Table 4).

**TABLE 4:** Results of the top 20 KEGG signaling pathways.

GO	Description	Enrichment	LogP	Count
hsa05200	Pathways in cancer	27.1909	−95.7693	81
hsa04151	PI3K-Akt signaling pathway	26.690	−61.2701	54
hsa05161	Hepatitis B	40.614	−48.6348	37
hsa05215	Prostate cancer	54.014	−46.4626	32
hsa05205	Proteoglycans in cancer	33.328	−46.405	38
hsa05417	Lipid and atherosclerosis	30.648	−43.7071	37
hsa04933	AGE-RAGE signaling pathway in diabetic complications	53.14546839	−43.2797	30
hsa05167	Kaposi sarcoma-associated herpesvirus infection	31.950	−41.9716	35
hsa04510	Focal adhesion	30.848	−41.3999	35
hsa04068	FoxO signaling pathway	41.703	−41.0224	31
hsa01521	EGFR tyrosine kinase inhibitor resistance	60.386	−40.688	27
hsa04218	Cellular senescence	36.468	−40.3057	32
hsa01522	Endocrine resistance	50.604	−39.6812	28
hsa05165	Human papillomavirus infection	20.417	−37.9125	38
hsa05163	Human cytomegalovirus infection	26.125	−36.472	33
hsa04010	MAPK signaling pathway	20.874	−35.1908	35
hsa04625	C-type lectin receptor signaling pathway	44.304	−35.1255	26
hsa04066	HIF-1 signaling pathway	42.290	−34.5372	26
hsa04015	Rap1 signaling pathway	26.163	−34.2501	31
hsa05230	Central carbon metabolism in cancer	57.960	−34.1623	23

### 4.4 Results of molecular docking

To further validate the predicted interactions between bioactive compounds of *I. hispidus* and key RCC-related targets, molecular docking simulations were performed. Five primary compounds—cerevisterol, (22E,24R)-ergosta-7,22-diene-3β,5α,6β,9α-tetrol, withanolide, Inonoterpene A, and polyporusterone D—were selected as candidate ligands based on their high degree centrality within the compound–target network. Correspondingly, five top-ranked core proteins from the PPI network—AKT1 (PDB ID: 1H10), β-catenin (CTNNB1, PDB ID: 2Z6H), epidermal growth factor receptor (EGFR, PDB ID: 1M14), signal transducer and activator of transcription 3 (STAT3, PDB ID: 6NJS), and B-cell lymphoma 2 (BCL2, PDB ID: 1G5M)—were chosen as receptor targets). The resulting binding energy scores are summarized in Supplementary Table S. Notably, withanolide displayed the most favorable docking scores across all targets, with particularly strong predicted interactions with EGFR (−9.3 kcal/mol) and BCL2 (−9.1 kcal/mol). For STAT3, our prediction (−8.8 kcal/mol) is consistent with prior experimental reports demonstrating direct binding of withaferin A to the STAT3 SH2 domain [Kim JH, Lee J, Singh SV. Withaferin A induces apoptosis and inhibits metastasis in human breast cancer cells by targeting vimentin and STAT3. Sci Rep. 2016; 6:27555. Zhang X, Samadi AK, Roby KF, et al. Inhibition of cell growth and induction of apoptotic cell death by withaferin A in human breast cancer cells. J Biol Chem. 2013; 288 (51):36555-36565.]. However, for cerevisterol, inonoterpene A, and polyporusterone D, no prior direct binding evidence exists, and these results should be interpreted as hypothesis-generating predictions requiring experimental confirmation.

**FIGURE 6:**
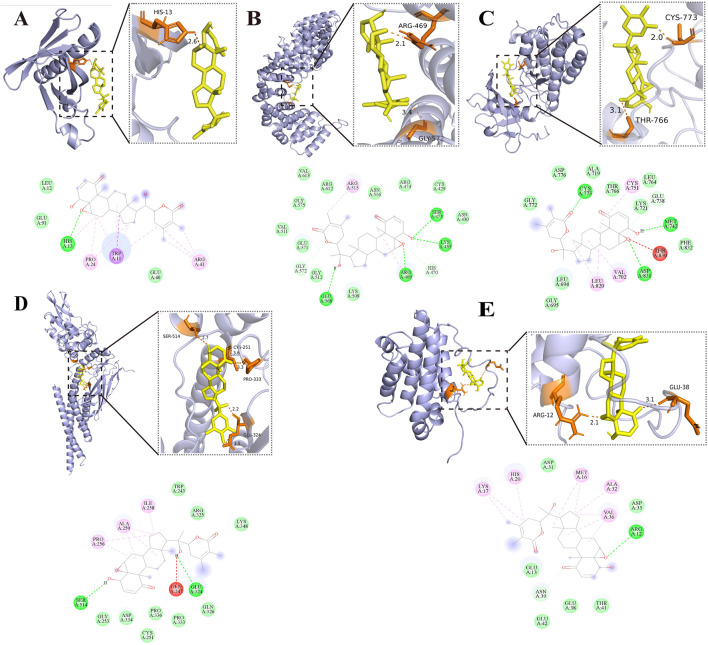
Molecular docking results of compounds and core targets. **(A)** withanolide-AKT1. **(B)** withanolide-CTNNB1; **(C)** withanolide-EGFR. **(D)** withanolide-STAT3. **(E)** withanolide-BCL2. (3D and 2D models).

The binding conformations of withanolide with each target protein were visualized using PyMOL and Discovery Studio (Figure 6). Figure 6A shows that withanolide binds to AKT1 via a hydrogen bond involving the HIS-13 residue. Figure 6B indicates that in CTNNB1, ARG-469 and GLY-572 participate in ligand binding. Figure 6C illustrates that binding to EGFR is mediated by TYR-773 and THR-766 at the active site. Figure 6D demonstrates that STAT3 interaction involves SER-514, GLY-251, PRO-333, and GLU-324. Figure 6E reveals that BCL2 binding is stabilized through contacts with ARG-12 and GLU-38.

### 4.5 Inonotus hispidus inhibits proliferation and migration of RCC

**FIGURE 7:**
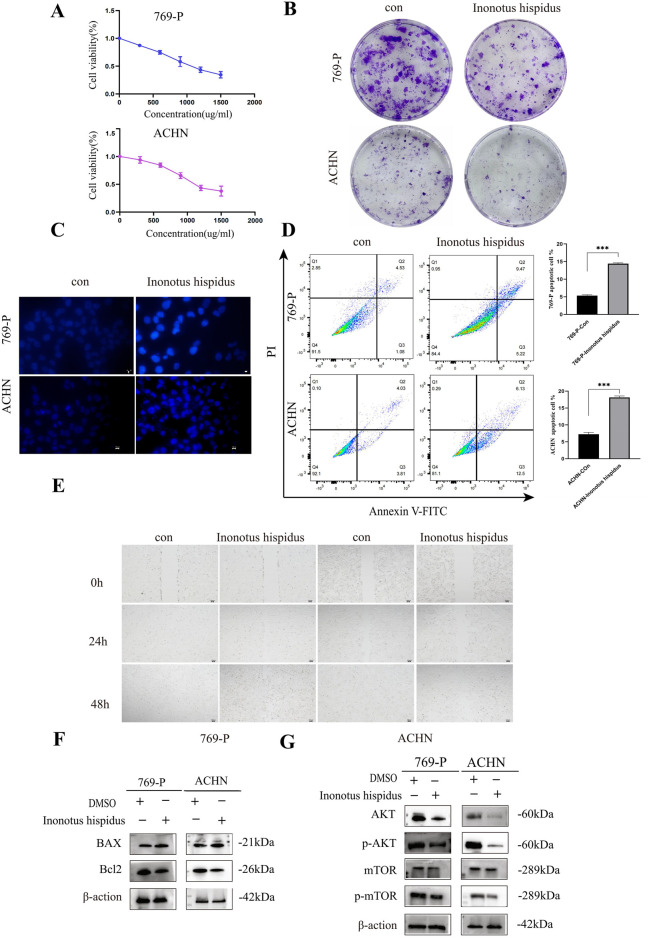
Inonotus hispidus inhibits RCC cell growth *in vitro*. **(A)** IC_50_ values were determined after treating 769-P and ACHN cells with various concentrations of EEIH for 24 h. **(B)** Colony formation assays of 769-P and ACHN cells treated with EEIH. **(C)** Fluorescence images and quantitative analysis of Hoechst-stained 769-P and ACHN cells treated with EEIH (1000 μg/mL) for 24 h. Scale bar: 50 μm. **(D)** Apoptosis of 769-P and ACHN cells analyzed by flow cytometry after 24 h of EEIH treatment. **(E)** Wound healing assays assessing the migration ability of 769-P and ACHN cells treated with EEIH (1000 μg/mL) for 24 and 48 h. Scale bar: 100 μm. **(F)** Western blot analysis of apoptosis- and proliferation-related protein expression in 769-P and ACHN cells treated with EEIH (1000 μg/mL). **(G)** Effects of EEIH on the expression of Akt, mTOR, p-Akt, and p-mTOR proteins in 769-P and ACHN cells.

To experimentally validate the antitumor function of *I. hispidus*, we assessed the effects of its ethanol extract (EEIH) on cell proliferation, apoptosis, and migration in human RCC cell lines 769-P and ACHN. A dose-related suppression of cell proliferation was detected following EEIH treatment, based on CCK-8 assay outcomes. The half-maximal inhibitory concentration (IC_50_) was approximately 1,000 μg/mL at 24 h (Figure 7A). This anti-proliferative effect was further corroborated by colony formation assays, where EEIH markedly reduced clonogenic survival over a 14-day period (Figure 7B). The EEIH-treated cells exhibited the apoptotic nuclear characteristics, such as chromatin condensation and nuclear fragmentation, as illustrated by Hoechst 33258 staining (Figure 7C). The apoptotic cell fraction was found to be significantly higher in the EEIH-treated group, based on flow cytometric analysis, which meant that the growth inhibition observed was related to the induction of apoptosis (Figure 7D). Moreover, wound-healing assays showed that EEIH significantly suppressed RCC cell migration at both 24 and 48 h post-treatment (Figure 7E). Molecular analysis through Western blot unveiled that EEIH-induced apoptosis is associated with Bax upregulation and Bcl-2 downregulation (Figure 7F). Collectively, significant *in vitro* antitumor effects of EEIH were observed, marked by reduced RCC cell proliferation and migration, along with increased apoptosis.

### 4.6 Inonotus hispidus suppresses the PI3K/Akt/mTOR pathway in RCC cells

The oncogenic progression of clear cell renal cell carcinoma (ccRCC) involves critical signaling events within the PI3K/Akt/mTOR pathway, which is often dysregulated by genetic mutations, copy number changes, and epigenetic mechanisms. These aberrations induce constitutive pathway activation in more than 70% of cases of ccRCC, which contributes to tumor initiation, progression, and reprogramming of metabolism, such as increased glycolysis, lipogenesis, and glutamine metabolism (Sato et al., 2013; Chakraborty et al., 2021). Consistent with predictions derived from network pharmacology analysis, a notable reduction in the phosphorylation states of Akt and mTOR was observed in EEIH-treated 769-P and ACHN cells, as revealed by Western blot (Figure 7G).

### 4.7 *In vivo* anti-RCC effect of Inonotus hispidus

**FIGURE 8:**
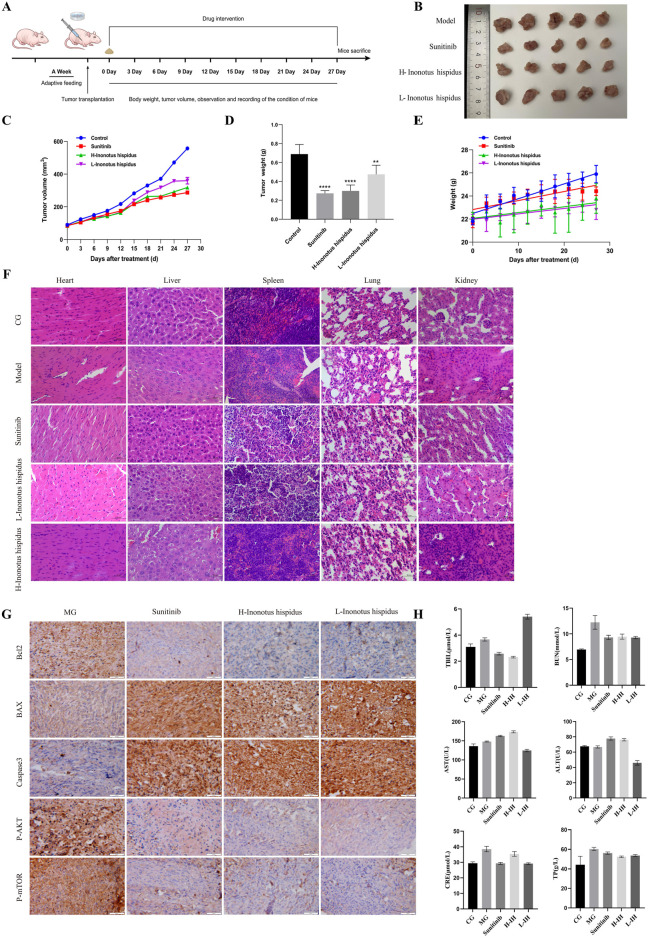
*Inonotus hispidus* exerts anti-RCC effects *in vivo*. **(A)** Schematic diagram of the workflow for Inonotus hispidus-mediated inhibition of subcutaneous tumor growth in BALB/c nude mice. **(B)** Images of excised tumors in different groups. **(C)** Representative images and growth curves of xenografts in nude mice (n = 5). **(D)** Weights of xenografts in nude mice (n = 5). **(E)** Body weight change curves of nude mice in each group throughout the observation period. Ns indicates no statistical significance. **(F)** Representative images of HE-stained visceral tissues (heart, liver, spleen, lung, kidney) from mice in each group at the end of the observation period. Scale bar: 50 μm. **(G)** Protein expression levels in tumor tissues assessed by IHC. **(H)** Serum biochemical indices in mice after different treatments. Serum levels of TBIL, BUN, AST, ALT, CRE, and TP were measured in each group of mice. Data are presented as mean ± SD (n = 5 per group). Statistical analysis was performed using one-way ANOVA. (Compared with the control group, **P < 0.01, ***P < 0.001, ****P < 0.0001, n = 5).

To assess the therapeutic potential and systemic safety of *I*. *hispidus in vivo*, a RCC xenograft model was established by subcutaneous inoculation of ACHN cells into female BALB/c nude mice. Mice were treated daily with low or high doses of the EEIH, with sunitinib serving as a positive control. As shown in Figures 8A–D, A clear dose-dependent antitumor response to EEIH was confirmed through significant reductions in tumor volume and terminal weight. Notably, the tumor-suppressive effect of high-dose EEIH was comparable to that observed in the sunitinib group. No measurable weight loss occurred in any group, and body weights remained stable throughout the dosing period (Figure 8E). No morphological abnormalities were identified by the histological examination of the heart, liver, spleen, lung, and kidney using H&E staining (Figure 8F). IHC assessment of tumor tissues (Figure 8G) revealed that proteins against programmed cell death, including Bcl-2, p-Akt, and p-mTOR, were downregulated in tumors, whereas the pro-apoptotic protein, BAX, and the executioner caspase, Caspase-3, were upregulated in the tumor tissues. The detected molecular changes imply that apoptosis occurred as a consequence of PI3K/Akt/mTOR signaling disruption. Collectively, these results point to the fact that EEIH can suppress the growth of RCC tumors *in vivo* and do so without apparent toxicity.

### 4.8 Serum biochemical indices indicate no obvious liver/kidney function parameters alterations of EEIH

To further evaluate systemic safety, serum biochemical markers of hepatic and renal function were assessed, including AST, ALT, TBIL, TP, BUN, and CRE (Figure 8H). Compared with the control and model groups, EEIH treatment did not show a consistent increase in liver injury markers (AST/ALT) or renal injury markers (BUN/CRE). Overall, these biochemical data, together with the stable body weight and the absence of apparent histopathological abnormalities in major organs, support that EEIH administration exhibited no obvious systemic hepatotoxicity or nephrotoxicity under the current dosing regimen.

## 5 Discussion

Network pharmacology, which is a branch of systems biology and network analytics, offers a solid platform on which the multi-component–multi-target–multi-pathway mechanisms in TCM formulations can be systematically explained. It allows predicting the therapeutic effects and potential toxicities comprehensively and mechanistically by integrating multi-omics data. This approach provides a mechanistic basis of discovering pharmacologically active compounds, target assessment and optimization of drug re-use plans. Consequently, network pharmacology has emerged as a fundamental paradigm in the scientific study of TCM and is involved in the modernization of traditional therapies and has gained broad acceptance within the pharmacological and translational research communities (Zhao et al., 2023). However, we have to acknowledge the inherent limitations of computer target prediction tools. SwissTarget Prediction relies on algorithms based on chemical similarity, which may result in false positives when structural features match known ligands without functional activity. Database bias may overestimate well studied targets, underestimate new interactions, and fail to consider tissue-specific expression or metabolic transformation. To alleviate these concerns, we implemented strict drug similarity filtering, cross validated predictions on five independent disease databases, applied network topology analysis to identify central targets, and most importantly, experimentally validated all computational predictions through molecular docking, *in vitro* detection, and *in vivo* models. Therefore, we generate network pharmacology results as hypotheses rather than final conclusions, and experimental validation is the main evidence of compound target relationships.

Through an integrative systems biology approach, this study revealed that *I. hispidus* engages multiple molecular targets and exerts anti-RCC effects by interfering with PI3K/Akt/mTOR signaling, as confirmed by computational and experimental validation. Among the 49 bioactive compounds identified through drug-likeness and gastrointestinal absorption screening, prioritization for experimental validation was guided by an integrated scoring system combining network pharmacology metrics with biological relevance. Specifically, compounds were ranked based on:network centrality, binding affinity (molecular docking scores against core targets Akt1, mTOR, PI3K), compound abundance and literature precedence. In this study,the major compounds included cerevisterol, (22E,24R)-ergosta-7,22-diene-3β,5α,6β,9α-tetrol, withanolide, Inonoterpene A, and polyporusterone D. emerged as top-ranked candidates due to their exceptional network connectivity (degree >15), favorable docking profiles against multiple PI3K/Akt/mTOR nodes, and documented anti-proliferative effects in renal cancer models. Cerevisterol has been reported to exert antitumor activity by modulating immune evasion, inflammatory signaling, and apoptosis. It acts through multiple pathways, including PD-1/PD-L1, NF-κB, PI3K/Akt, ERBB/EGFR, and NK cell–mediated cytotoxicity, and has demonstrated anti-lymphoma effects in combination with other active components (Jin et al., 2024). Cytotoxicity screening revealed that cerevisterol from *Pleurotus nebrodensis* possesses moderate anticancer activity against MCF-7 cells (Hao et al., 2017). Withanolide compounds are known for their multi-target anti-cancer activity, regulating autophagy, apoptosis, and ferroptosis, while also inhibiting signaling pathways associated with tumor metastasis (Chen et al., 2023; Jung et al., 2022). Polyporusterone D, a steroidal derivative of *Polyporus umbellatus*, has exhibited *in vitro* cytotoxicity by preventing the proliferation of tumor cells (Ohsawa et al., 1992). Inonoterpene A is a lanostane-type triterpenoid, which is isolated from *I. hispidus*, possessing neurotrophic, anti-inflammatory, and antioxidant activity, and contributes to the overall pharmacological profile of the extract (Kou et al., 2021). This multi-criteria ranking approach ensured that selected compounds represented both computationally predicted key players and biologically plausible therapeutic agents, thereby strengthening the translational relevance of our experimental validation.

It was found that there are 169 overlapping genes that are potential therapeutic targets of *I. hispidus* in RCC, which raises the question of whether this reflects broad-spectrum therapeutic potential or limited specificity. The PPI network analysis identified AKT1, CTNNB1, EGFR, STAT3 and BCL2, which occupied central roles in important oncogenic signaling pathways that are central to the pathogenesis of RCC, especially ccRCC. We propose a balanced interpretation: RCC involves multiple dysregulated pathways (PI3K/Akt/mTOR, VEGF/HIF, JAK/STAT), and multi-target modulation may overcome compensatory mechanisms, as evidenced by FDA-approved RCC drugs (sunitinib, sorafenib, cabozantinib) exhibiting similar multi-target profiles. However, we acknowledge that not all 169 targets are RCC-specific. Our network analysis identified core hub targets (AKT1, EGFR, STAT3, BCL2, mTOR, VEGFA) representing established RCC drivers. We conclude this reflects a ‘selectively multi-target’ profile rather than non-specific pan-activity, with convergence on RCC drivers suggesting therapeutic potential while acknowledging unequal target contributions. Future RCC-specific models and biomarker stratification will distinguish RCC-specific versus general antitumor effects. AKT1, which is one of the key players in PI3K/Akt/mTOR signaling, is significantly upregulated in the RCC tissues compared to the adjacent normal kidney tissue. Its activation is linked to reduced WHO/ISUP tumor grades and leads to malignant progression by enhancing cell proliferation, migration, invasion, resistance to apoptosis, and G0/G1 cell cycle arrest (Li Z. et al, 2022; Choi et al., 2022). CTNNB1 is also dysregulated in RCC with increased mRNA and protein levels, hypomethylation, and phosphorylation at serine residues S675 and S191, which indicates its potential to serve as a diagnostic biomarker (Xu et al., 2024). EGFR, commonly hyperactivated in RCC as a consequence of disrupted regulatory axes (MIAC-AQP2 and MFN2-Rab21-PTPRJ), contributes to improved tumor cell proliferation, metastasis, and survival, and is linked to negative clinical outcomes (Luo et al., 2023; Li M. et al, 2022). The activation of STAT3 by the MIAT/JAK3 signaling axis, triggers oncogenic transcription through downstream molecules like cyclin D1 and Myc, which consequently facilitate the progression of the cell cycle and promotes the capacity to metastasize. The anti-apoptotic protein, BCL2, is increased in both the primary (A-498) and metastatic (Caki-1) RCC models. It is noteworthy that simultaneous BCL2 and mTOR inhibition improves the antitumor activity of everolimus, which adds weight to its role as a co-targeted agent in the PI3K/Akt/mTOR axis (Nayman et al., 2019). These findings were further supported by molecular docking analysis since they showed high binding affinities between the five core targets and the major bioactive compounds of *I. hispidus*, that is, cerevisterol, (22E,24R)-ergosta-7,22-diene-3β,5α,6β,9α-tetrol, withanolide, Inonoterpene A, and polyporusterone D. Among them, withanolide had the most desirable binding energy across all core targets. Although our data demonstrate clear downregulation of p-Akt and p-mTOR, we acknowledge that this study does not fully exclude contributions from parallel signaling pathways such as MAPK/ERK or JAK/STAT3. Previous studies have shown crosstalk between these pathways. Future studies employing pathway-specific inhibitors and rescue experiments will be needed to definitively establish the relative contributions of each pathway.

Key metabolic activities such as protein and nucleotide biosynthesis, glucose uptake, lipid utilization, and autophagic flux are regulated by the PI3K/Akt/mTOR signaling pathway. It also regulates essential processes like cell growth, angiogenesis, survival and proliferation. In RCC, pathological activation of this pathway is involved in sustaining tumor cell viability, promoting proliferation, enhancing migration, and enabling metastasis (Miricescu et al., 2021). In line with these results, KEGG pathway enrichment analysis of this study revealed PI3K-Akt signaling cascade as the most highly enriched pathway among the overlapping targets of *I. hispidus*. Based on this forecast, experimental confirmation showed that the EEIH had a potent effect on preventing RCC cell proliferation, colony formation, and migration *in vitro*, causing apoptosis and decreasing the phosphorylation of Akt and mTOR. The above effects were also confirmed *in vivo* on an RCC xenograft mouse model, where EEIH treatment suppressed tumor growth efficiently, as indicated by volume and weight measurements, and exhibited a favorable safety profile without systemic toxicity. IHC analysis of tumor tissues revealed downregulation of Bcl-2, p-Akt, and p-mTOR, alongside, accompanied by upregulation of Bax and Caspase-3. All these data prove EEIH achieves its antitumor action by suppressing the PI3K/Akt/mTOR signal transduction and triggering the mitochondrial apoptotic cascade. The concentration of EEIH is approximately 1000 μg/mL compared to a single compound standard, with relatively high *in vitro* IC50. However, this is consistent with the research on crude extracts of traditional Chinese medicine, in which the synergistic effect of multiple components rather than single molecule potency drives the therapeutic effect. The feasibility of transformation is supported by our *in vivo* data, which shows that at a dose of 200 mg/kg, tumors are significantly inhibited without toxicity, indicating that systemic exposure requirements may differ from *in vitro* conditions. In addition, considering the traditional oral administration of *I. hispidus*, the local gastrointestinal concentration after oral administration may exceed plasma levels. In this context, bioaccumulation enhancement strategies (such as nanoformulations) or combination therapy with existing RCC drugs may be necessary for clinical translation. Pharmacokinetic studies measuring plasma and tumor concentrations in the future are crucial for determining clinically achievable exposure levels. In addition, an important gap exists between extract-based effects and purified compound activity. While multi-component synergy may enhance efficacy, it complicates mechanistic attribution, regulatory approval, and batch standardization. Future studies should employ fractionation to identify active constituents, evaluate single versus combined compounds, and develop marker-based standardization methods. Despite these limitations, the convergence of our *in vitro*, *in vivo*, and computational findings provides robust preliminary evidence warranting further investigation in clinically predictive models (PDX, orthotopic, humanized mice) and formal toxicology/pharmacokinetic studies.

While several TCM-derived compounds have been reported to modulate the PI3K/Akt/mTOR pathway in RCC, including ginsenosides from Panax ginseng and ganoderic acids from Ganoderma lucidum, our study provides distinct advances beyond existing literature. First, this is the first systematic investigation of *I. hispidus*-derived phenolic compounds (particularly hispidin derivatives) as multi-target agents against ccRCC, expanding the repertoire of medicinal fungi with demonstrated anti-RCC activity beyond the more extensively studied Ganoderma and Cordyceps species. Second, unlike previous network pharmacology studies that rely solely on computational predictions, we provide comprehensive experimental validation across both *in vitro* and *in vivo* models, establishing causal relationships between predicted compound. Third, our identification of specific hispidin derivatives as key bioactive constituents with superior binding affinities to mTOR and Akt1 represents a novel finding that warrants further drug development investigation. Finally, the demonstration that *I. hispidus* extracts achieve anti-tumor efficacy at clinically relevant doses without significant toxicity, combined with their potential to synergize with current RCC therapies, suggests translational applications in overcoming therapeutic resistance-a critical unmet need in advanced RCC management. However, several translational challenges remain. First, extract standardization is critical, as variations in culture conditions and extraction methods impact bioactive compound composition. Implementation of GMP-compliant protocols with fingerprinting techniques (HPLC, LC-MS) will ensure batch-to-batch consistency. Second, inter-batch variability requires establishment of marker compound criteria, stability studies, and quality control measures. Advances in fungal genomics and metabolomics offer opportunities to optimize production, while academic-industry-regulatory collaboration will be essential for successful bench-to-bedside translation. Collectively, these contributions advance the field beyond correlative associations toward mechanistically grounded therapeutic development.

## 6 Conclusion

To illustrate how *I. hispidus* exerts its antitumor effects in RCC, this study used an integrative methodology that incorporated network pharmacology, molecular docking, *in vitro* and *in vivo* experimental validations. The observed inhibition of proliferation, colony formation, and migration, alongside increased apoptosis, indicates that *I. hispidus* has potent anticancer activities. Mechanistically, these are mediated by the inhibition of PI3K/Akt/mTOR signaling pathway. Bioinformatic analyses identified five core molecular targets, AKT1, CTNNB1, EGFR, STAT3, and BCL2, with key bioactive compounds, including cerevisterol, (22E,24R)-ergosta-7,22-diene-3β,5α,6β,9α-tetrol, withanolide, Inonoterpene A, and polyporusterone D, demonstrating high-affinity binding interactions. *In vivo* experiments using an RCC xenograft model confirmed the therapeutic efficacy of EEIH, which resulted in a significant reduction of tumor burden without inducing systemic toxicity. Together, these results highlight *I. hispidus* as a promising multi-target natural agent with potential for development into a clinically relevant therapeutic for RCC. Although this study demonstrates the antitumor potential of EEIH in RCC through integrated network pharmacology, molecular docking, and experimental validation, several limitations should be acknowledged: target predictions rely on *in silico* approaches without direct biochemical binding validation; molecular docking results require experimental confirmation; *in vivo* findings are based on a single xenograft model lacking immune components; and relative contributions of individual extract constituents remain undefined. Systematic follow-up studies addressing these recommendations will be essential to translate EEIH from promising preclinical candidate to clinically viable RCC therapeutic.

## Data Availability

The original contributions presented in the study are included in the article. Further inquiries can be directed to the corresponding author.
