# Supplementary material for: Mechanism of Inonotus hispidus in suppressing renal cell carcinoma proliferation via regulation of the PI3K/AKT/mTOR pathway
Source: Front Pharmacol. 2026 May 12;17:1773406. doi: 10.3389/fphar.2026.1773406 (PMC13201449; doi:10.3389/fphar.2026.1773406)
Supplement: Supplementary file 1 [file Supplementaryfile1.docx]

**Table S**

The binding energy of compounds and core targets (kcal/mol).

| **Chemical component** | **Binding energy(kcal/mol)** | | | | |
| --- | --- | --- | --- | --- | --- |
|  | AKT1  PDB ID  1H10 | CTNNB1  PDB ID  2Z6H | EGFR  PDB ID 1M14 | STAT3  PDB ID 6NJS | BCL2  PDB ID  1G5M |
| cerevisterol | -6.7 | -7.6 | -8.5 | -7.5 | -7.8 |
| (22E,24R)-ergosta-7,22-diene-3β,5α,6β,9α-tetrol | -6.7 | -7 | -8.3 | -7.7 | -7.3 |
| withanolide | -8 | -8.5 | -9.3 | -8.8 | -9.1 |
| Inonoterpene A | -6.5 | -7.5 | -8.2 | -7.9 | -7.4 |
| Polyporusterone D | -6.9 | -7.9 | -7.9 | -7.9 | -7.2 |
